# Different roles of alpha and beta band oscillations in anticipatory sensorimotor gating

**DOI:** 10.3389/fnhum.2014.00446

**Published:** 2014-06-17

**Authors:** Verena N. Buchholz, Ole Jensen, W. Pieter Medendorp

**Affiliations:** ^1^Cognition and Behaviour, Donders Institute for Brain, Radboud University NijmegenNijmegen, Netherlands; ^2^Department of Neurophysiology and Pathophysiology, University Medical Center Hamburg-EppendorfHamburg, Germany

**Keywords:** human, MEG, parietal cortex, sensorimotor, gating, reference frame

## Abstract

Alpha (8–12 Hz) and beta band (18–30 Hz) oscillations have been implicated in sensory anticipation and motor preparation. Here, using magneto-encephalography, we tested whether they have distinct functional roles in a saccade task that induces a remapping between sensory and motor reference frames. With a crossed hands posture, subjects had to saccade as fast and accurate as possible toward a tactile stimulus delivered to one of two non-visible index fingers, located to the left or right of gaze. Previous studies have shown that this task, in which the somatotopic stimulus must be remapped to activate oculomotor system in the opposing hemisphere, is occasionally preceded by intrahemispheric remapping, driving a premature saccade into the wrong direction. To test whether the brain could anticipate the remapping, we provided auditory predictive cues (80% validity), which indicated which finger is most likely to be stimulated. Both frequency bands showed different lateralization profiles at central vs. posterior sensors, indicating anticipation of somatosensory and oculomotor processing. Furthermore, beta band power in somatosensory cortex correlated positively with saccade reaction time (SRT), with correlation values that were significantly higher with contralateral vs. ipsilateral activation. In contrast, alpha band power in parietal cortex correlated negatively with SRT, with correlation values that were significantly more negative with ipsilateral than contralateral activation. These results suggest distinct functional roles of beta and alpha band activity: (1) somatosensory gating by beta oscillations, increasing excitability in contralateral somatosensory cortex (positive correlation); and (2) oculomotor gating by posterior alpha oscillations, inhibiting gaze-centered oculomotor regions involved in generating the saccade to the wrong direction (negative correlation). Our results show that low frequency rhythms gate upcoming sensorimotor transformations.

## Introduction

Saccadic eye movements serve to bring objects of interest into our focus. To make these movements, the object’s sensory coordinates must be converted into gaze-based oculomotor coordinates. For visually-guided saccades, this transformation is fairly straightforward because the visual and motor coordinates are the same (Andersen and Buneo, 2002). In contrast, making saccades toward something felt on the skin, e.g., to inspect the insect landed on your hand, involves a more complex transformation. In this case, the tactile information, as sensed in a body-based somatotopic frame (i.e., relative to the body’s surface), must be transformed into the oculomotor representation, which depends on the position of both hand and gaze (Groh and Sparks, 1996; Ren et al., 2006; Azañón et al., 2010; Harrar and Harris, 2010). What are the neural implications?

Because the body-based somatosensory and gaze-centric oculomotor maps are lateralized in the cortical brain (Medendorp et al., 2003; Eickhoff et al., 2008), these transformations sometimes require interhemispheric remapping. For instance, when the right hand (RH) is to the left of gaze (in the left visual hemifield), the tactile stimulus is sensed in a somatosensory map in the left hemisphere, but must be remapped to an oculomotor representation in the right hemisphere. Previous studies suggest that early somatotopic processing activates the oculomotor system in the same hemisphere, occasionally even strong enough to drive a saccade into the wrong direction (Overvliet et al., 2011; Buchholz et al., 2012). To account for the integration of postural information in the sensorimotor transformation, activity must build up on the other side of the oculomotor system, and by superseding the erroneous activity, it could initiate a saccade into the correct direction. But this process takes time and delays the saccade. Therefore, it would be beneficial if the brain could anticipate the upcoming sensorimotor transformation and regulate which regions need to be engaged and disengaged (Jensen and Mazaheri, 2010), even before the stimulus arrives. So far, these anticipatory mechanisms have been identified for sensory, as well as motor processes independently, but never in the context of a sensorimotor task, requiring spatial transformations. Importantly, such mechanisms across spatial maps could not only be important for sensorimotor behavior, but could also be instrumental in supra-modal attention networks.

A mechanism that has been proposed to reflect gating is low-frequency oscillatory activity. Relative power suppression in the alpha (10 Hz) and beta-band (18–30 Hz), which is linked to cortical excitability during sensory and motor tasks (Gilbertson et al., 2005; Romei et al., 2008, 2010; Engel and Fries, 2010; Haegens et al., 2011b; Jensen et al., 2012; van Ede et al., 2012) has been shown to correlate with various perceptual benefits, including faster and better detection in tactile and visual tasks (Thut et al., 2006; van Ede et al., 2010, 2012; Haegens et al., 2011a; Händel et al., 2011). Both rhythms show lateralization with less power in the contralateral than ipsilateral respective sensory cortex in anticipation of visual or tactile stimuli (Worden et al., 2000; Thut et al., 2006; Hanslmayr et al., 2007; Jensen and Mazaheri, 2010; Haegens et al., 2011a; van Ede et al., 2011; Bauer et al., 2012).

In all these visual and tactile studies, the low-frequency power suppression involved the same hemisphere (van Ede et al., 2011; Bauer et al., 2012). Does this mean that alpha and beta band generally play similar roles in gating? This conclusion could be premature. In these studies, tactile tasks were typically performed with the hands in natural, uncrossed position, while the visual tasks were tested with gaze fixating straight ahead. This means that the power modulations, which were observed in the same hemisphere, were not functionally dissociated in terms of the reference frame that they deploy.

In the present study we used a crossed hand position to examine the lateralization profiles of alpha and beta band power while subjects anticipate the tactile remapping for a saccade. Recently, we reported alpha and beta band power modulations in body- and gaze-centered reference frames induced by a tactile stimulus for a saccade (Buchholz et al., 2011). Here we test whether these oscillations also prepare the brain for upcoming tactile remapping in these different frames, thereby setting a gate for sensorimotor behavior at different stages of the sensorimotor transformation.

Under continuous recording of magnetoencephalography (MEG), human subjects executed speeded saccades to tactile stimuli (predictively cued with 80% validity) for which correct saccades require interhemispheric remapping. If alpha and beta oscillations play a role in anticipating tactile remapping for saccades, their modulations should (1) not only be evident in body-based somatotopic but also in gaze-centered oculomotor structures, even if the prediction is probabilistic; (2) take position of the hand relative to gaze into account; and (3) facilitate tactile remapping for saccades.

## Materials and methods

### Participants

Twenty-two subjects (age range 19–50 yrs, 12 female, 3 left handed), free of any known sensory, perceptual, or motor disorders, volunteered to participate in the experiment. All subjects provided written informed consent according to institutional guidelines of the local ethics committee (CMO Committee on Research Involving Human Subjects, region Arnhem-Nijmegen, the Netherlands).

### Setup

Participants sat in the MEG system that was placed in a magnetically shielded dark room. They wore ear tubes attached to earplugs for auditory instructions. Their elbows were resting on platform in front of them. Forearms were crossed at the level of the wrists and supported by a wooded board, pitched away by 30° relative to the subject’s body. In this configuration the hands were about 25 cm in front of the body. The index fingers were stretched. Due to the crossed hands posture, the two index fingertips were positioned 10 cm contralateral relative to the body midline (i.e., the sagittal plane).

One fiber optic light (Omron e3x-na, GB) was located just above the center between the two hands and served as a fixation point. Subjects viewed this light with a comfortable, slightly downward gaze direction.

We induced a tactile stimulus by means of electrical stimulation (single pulse, duration 200 μs) of the nerve endings in the skin of either index fingertip. The simulation was applied using two constant-current high voltage stimulators (Digitimer Ltd., Hertfordshire, UK). Stimulus intensity was set beyond individual perceptual threshold, but below pain threshold. Stimulus levels were adjusted during the experiment to account for adaptation effects of the tactile sense.

Continuous MEG data were recorded using a whole head system with 275 axial gradiometers (Omega 2000, CTF Systems Inc., Port Coquitlam, Canada). Head position relative to the sensor array was measured using localization coils fixed at anatomical landmarks (nasion, and left and right ear). Horizontal and vertical electrooculograms (EOG) were recorded using electrodes placed below and above the left eye and at the bilateral outer canthi. Impedance of all electrodes was kept below 5 kΩ. During the experiment, eye recordings were continuously inspected to ensure the subject was vigilant and performed the task correctly. MEG and EOG signals were low-pass filtered at 300 Hz, sampled at 1200 Hz, and then saved to disk.

Structural full-brain MRIs were acquired with a 1.5 T Siemens Sonata scanner (Siemens, Erlangen, Germany) using a standard T1-weighted scan sequence (*FA* = 15°; voxel size: 1.0 mm in-plane, 256 × 256, 164 slices, *TR* = 760 ms; *TE* = 5.3 ms). These anatomical MRIs were recorded with anatomical reference markers at the same locations as the head position coils during the MEG recordings. The reference markers served alignment of the MEG and MRI coordinate systems.

### Experimental paradigm

Subjects performed a speeded response task in the dark, in which they had to saccade toward a tactile stimulus, delivered to one of the invisible index fingertips. Each trial began with the presentation of a high or low pitch tone, indicating with 80% validity which index finger was to be stimulated. Prior to the experiment, subject learned this relationship, which was counterbalanced across subjects. While the subject fixated centrally, after a 1.3–1.6 s interval the stimulus was delivered. Subjects were instructed to change their gaze as fast and accurate as possible to the invisible target location. After a brief fixation, the auditory cue of the next trial, instructed them to return to central fixation again. Subjects performed 10 blocks of 100 trials each, in which target locations were pseudo-randomly interleaved. Each trial lasted for 3000–3300 ms. A brief rest was provided between the blocks during which the subjects could move their hands and eyes freely.

**Figure 1:**
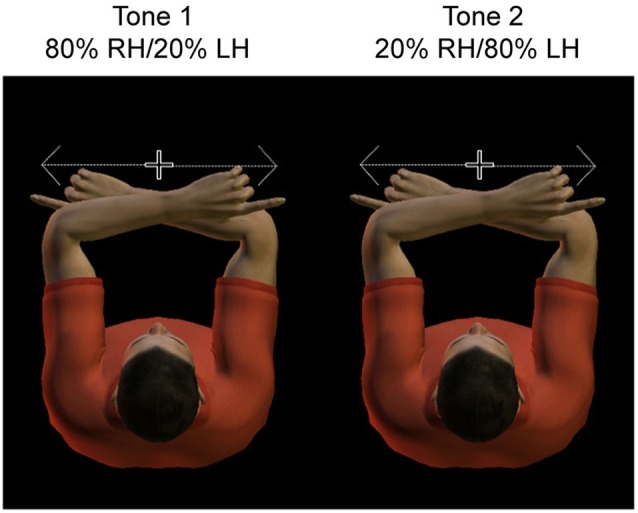
**Experimental design.** Subjects adopted a crossed hand posture, with the index fingers each at 10 cm distance from straight ahead. They had to fixate centrally, at a dim light between the two hands. Hands were resting on a tilted support, such that fixation was only slightly downward. Subjects had to saccade as fast and accurate as possible toward the tactile stimulus presented to the invisible fingertip. A tone cued with 80% validity the side of stimulation, such that subjects could anticipate the location of the sensory stimulus.

Thus the paradigm contains valid trials, in which the actual tactile stimulus location matches the expected location, and invalid trials, in which the actual tactile stimulus location is diametrically opposite from the expected location. Figure 1 illustrates the conditions of the paradigm, which are defined by the location of the target relative to the body (left vs. right hand) and cue validity (valid vs. invalid). That is, the location of the potential tactile target could be represented to the body, Left hand (LH) vs. right hand (RH), or alternatively, right or left relative to gaze. Due to the crossed hands posture, the hemisphere contralateral to the hand is ipsilateral to the target relative to gaze, and vice versa.

The present paper is based on the well-accepted notion in the literature that alpha and beta suppression in rolandic and posterior regions reflect cortical increased excitability (Gilbertson et al., 2005; Romei et al., 2008, 2010; Engel and Fries, 2010; Haegens et al., 2011b; Jensen et al., 2012; van Ede et al., 2012). Therefore, by dissociating relative suppression in the hemisphere contralateral to the hand or contralateral to the target in gaze coordinates, we can distinguish between gaze- and body-centered reference frames in the regions that anticipate the tactile remapping for the saccade.

### Behavioral analysis

Trials were rejected if subjects broke fixation 500 ms prior to stimulus presentation, as identified by semi-automatic analysis. On average, 74 ± 33 trials out of 1000 were excluded from further analysis. Supporting the effectiveness of this rejection method, all the reported effects show a topographic distribution that is inconsistent with residual saccadic eye movement contamination, as it is described extensively for (micro-) saccades in Carl et al. (2012) for MEG data. Of the included trials, saccade behavior was classified as “correct” when subject responded with a saccade into the correct direction after stimulus presentation. Trials were characterized as “error trials” when the saccade was initiated into the wrong direction, even when corrected during the movement. Trials that were classified as premature or too slow due to lack of subject alertness (RTs < 50 or > 450 ms, respectively) or trials in which subjects did not made a saccade at all were excluded. Based on these criteria, per subject 606 ± 45 valid trials, and 130 ± 14 invalid trials were correctly performed. We determined the reaction time of these correct saccades using a computer algorithm that detects a two degrees difference to fixation values, on a trial by trial basis.

### MEG data analysis

Open source Fieldtrip software^1^ (Oostenveld et al., 2011) was used to analyze the MEG data. Planar gradient estimation was calculated from the axial gradiometer signals using the nearest-neighbor method described by Bastiaansen and Knösche (2000) to simplify interpretation of the sensor-level data. With this conversion, the maximal signal is located above the source (Hämäläinen et al., 1993). The sum of the calculated horizontal and vertical planar MEG field gradients was computed to obtain the power at each virtual planar gradiometer location. Semi-automatic artifact rejection was done, rejecting high noise levels in MEG data by identifying outliers when calculating variance per trial.

Low frequency analysis (2–40 Hz) was computed based on a Fourier approach, applied to the 500 ms interval before stimulation and a Hanning taper, resulting in a spectral smoothing of approximately 3 Hz. Frequency bands of interest were the alpha band (10 ± 2 Hz) and the beta band (18–30 ± 2 Hz). To reduce data dimensionality and increase sensitivity of the analysis, we defined sensor clusters of interest based on previous results on tactile remapping, as reported by Buchholz et al. (2013), overlaying somatosensory (“central”) and posterior parietal (“posterior”) regions.

At the sensor level, we computed the pre-stimulus changes in power in the two frequency bands comparing activity in the contralateral and ipsilateral hemisphere for each condition. This analysis of pre-stimulus power was performed on a trial-by-trial basis, involving log-transformed power values of the last 500 ms preceding the stimulus in order to be as temporally close as possible to the transformation process. Both valid and invalidly cued trials were incorporated since subjects had the same expectancy during the pre-stimulus period, irrespective of trial type. To increase signal-to-noise ratio, we pooled this hemispheric difference across conditions and projected it onto a left standardized hemisphere, as in Buchholz et al. (2013). This spatially specific lateralization was compared across central and posterior sensors for both frequency bands separately with a simple *t*-test across subjects.

In a subsequent analysis, we correlated pre-stimulus activity with saccadic reaction time (SRT) of the correct saccades of the valid trials. To allow this relationship to be nonlinear, we calculated the Spearman rank correlation between log-transformed power values and SRT, only including validly cued trials. These correlation values were then Fisher *z*-transformed. Statistical effects were tested using paired *t*-tests.

To reconstruct the neural sources of the spectral components of interest at source level, we applied an adaptive spatial filtering (or beamforming) technique (Dynamic Imaging of Coherent Sources (DICS; Gross et al., 2001; Liljestrom et al., 2005)). We divided each participant’s brain volume into an individually spaced three-dimensional grid using SPM8,^2^ in which each location corresponds to a location in the regular 1 cm grid based on a brain template (International Consortium for Brain Mapping; Montreal Neurological Institute (MNI), Montreal, Canada). Then individual MRIs were warped to fit this template MRI and the template’s grid. We subsequently warped the grid back to fit every subject’s original MRI to obtain a grid in MNI coordinates for each subject. This procedure does not require normalization, as grid points are comparable across subjects. The individual spatial filters were computed from forward models with respect to dipolar sources at each individual grid point (the leadfield matrix) and the cross spectral density between all combinations of sensors at the frequency of interest (Nolte, 2003). This filter fully passes activity from the location of interest, while attenuating activity from all other locations (Van Veen et al., 1997). We used single-sphere head models based on individual MRIs to calculate the lead field matrix (Nolte, 2003). For every single subject, the source power was estimated relative to the source power in the other hemisphere, without the use of a baseline interval. Individual trial source power was log transformed and averaged across trials before averaging across subjects.

## Results

### Behavioral analysis

With their arms in a crossed posture, subjects performed a speeded saccade task to tactile stimuli, presented to the fingertips. Auditory predictive cues indicated with 80% validity which finger is most likely to be stimulated. For the valid trials, mean saccadic reaction time (SRT) of correct responses did not differ between stimuli presented to the left and right hand (*t*-test, *P* > 0.05). Average SRT in valid trials (256 ± 9 ms; Mean ± SD) was significantly shorter (*t*-test, *P* < 0.05) compared to invalid trials (263 ± 9 ms), i.e., the trials with the unexpected stimulus location. This validates our design, indicating that subjects used the auditory cues to anticipate the most probable stimulus location. Furthermore, percentage error trials for expected and unexpected LH stimuli were 3 and 6%; percentage error trials for expected and unexpected right hand stimuli were 4 and 9%. Given the low number of trials in the unexpected condition, in the following section, we will focus on the power modulation during the valid trials only.

### Low-frequency power modulations in sensory and motor frames

By design of the paradigm, the crossed posture imposes an interhemispheric remapping of tactile stimuli between the body-based somatosensory and gaze-centric oculomotor maps. We describe the lateralization of our two frequency bands of interest (alpha, beta) at the sensors of interest (central and posterior) during the prestimulus period. Under the assumption that relative suppression of alpha and beta oscillations reflects increased cortical excitability, and conversely that relative enhancement reflects cortical inhibition, we dissociated the hemispheric lateralization of these rhythms in terms of body-centered or gaze-centered anticipation.

**Figure 2:**
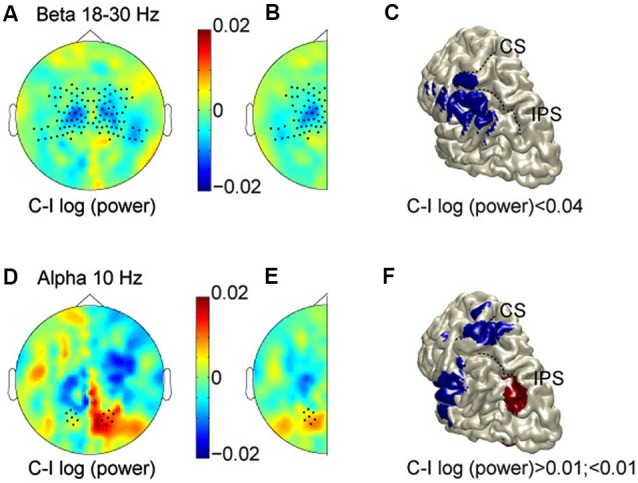
**Prestimulus modulations in alpha and beta bands. (A)** Scalp topography in the beta band (averaged across 18–30 Hz, and at time −0.3 s). Cooler colors, lower power for anticipating contralateral hand stimuli; warmer colors, lower power for stimuli on the ipsilateral hand. **(B)** Data combined across hemispheres. **(C)** Source reconstruction of the relative beta suppression contralateral to the expected hand stimulation. **(D–F)** Scalp topography and source reconstructions of the alpha oscillations (10 Hz, and time −0.3 s) in the same format as A–C. CS, Central sulcus; IPS, intraparietal sulcus.

Figure 2A shows the scalp topography of power in the beta band (averaged across 18–30 Hz) in the 500 ms prestimulus period, comparing log-transformed power when subjects were expecting a stimulus on the contralateral hand as compared to the ipsilateral hand. Thus, for the left hemisphere we compare right-hand (RH)—LH stimulation, and for the right hemisphere: LH—RH. Regions with cooler colors indicate lower power values for anticipating contralateral hand stimuli, while regions with warmer colors signify lower power values for stimuli on the ipsilateral hand. The scalp topography shows lower beta-band power for contralateral hand stimuli than for ipsilateral stimuli (cooler colors), most prominently over central regions. This is consistent with increased excitability in the hemisphere contralateral to the hand in a body-centered (somatotopic) representation format, or a decreased excitability in the hemisphere ipsilateral to the hand. To examine consistent effects across hemispheres, and improve the signal-to-noise ratio, data were combined by averaging across the two halves, resulting in a cleaner topography of the lower power for anticipated stimuli to the contralateral hand (Figure 2B). As shown, opposite modulations across hemispheres, which are inconsistent with either reference frame, and just reflect a general spatial bias have cancelled out. The observed lateralization was significantly different between central and posterior sensors (indicated by dots; *t* = 3.55, *P* = 0.0019). In fact, there was a clear lateralization at central sensors, but not at posterior sensors, which is consistent with sensory anticipation at central regions by beta band activity in a somatotopic reference frame.

We used spatial filtering techniques to estimate the sources underlying these anticipatory power changes, which are projected on a rendered representation of a standardized left hemisphere (Figure 2C). This suggests that the somatotopic pre-stimulus power modulation in the beta band originate from somatosensory areas, extending into inferior parietal cortex.

Whereas these body-centered modulations in somatosensory areas support previous findings, the crucial question here is whether the anticipation exceeds the sensory (somatotopic) level, and accounts for the transformations needed to operate at the motor level. In other words, does the brain also anticipate the gaze-centered motor representation of the potentially upcoming stimulus, taking into account the posture configuration between body and gaze? Or, in terms of topography, is there evidence for higher power values contralateral to the target in body-coordinates (warmer colors), corresponding to lower power values contralateral to the target in gaze-coordinates?

The beta band did not show any gaze-centered modulation at central or parietal sensors, i.e., relative power suppression contralateral to the saccade direction. The alpha band, however, showed a different pattern. Figures 2D–F plots alpha band topography when subjects were expecting a stimulus on the contralateral vs. the ipsilateral hand, in the same color format as Figures 2A–C. Lateralization of alpha-band power lateralization was significantly different between central and posterior sensors (*t* = −2.61, *P* = 0.01), by showing opposite modulation profiles. At the central sensors, the alpha band modulations mimic those of the beta band. At posterior sensors, mean alpha power is relatively higher when expecting contralateral compared to ipsilateral hand stimuli, consistent with a relative suppression contralateral to the target in gaze-coordinates. Furthermore, along the posterior midline, alpha band activity shows more power for left than for right stimuli, in both hemispheres, which is inconsistent with either reference frame, as seen in Figure 2E. Finally, the alpha band activity that is lateralized like the beta band, seems to originate from the hand region of primary somatosensory cortex and the operculum, whereas the opposite lateralization profile is observed in posterior intraparietal sulcus (pIPS; Figure 2F).

**Figure 3:**
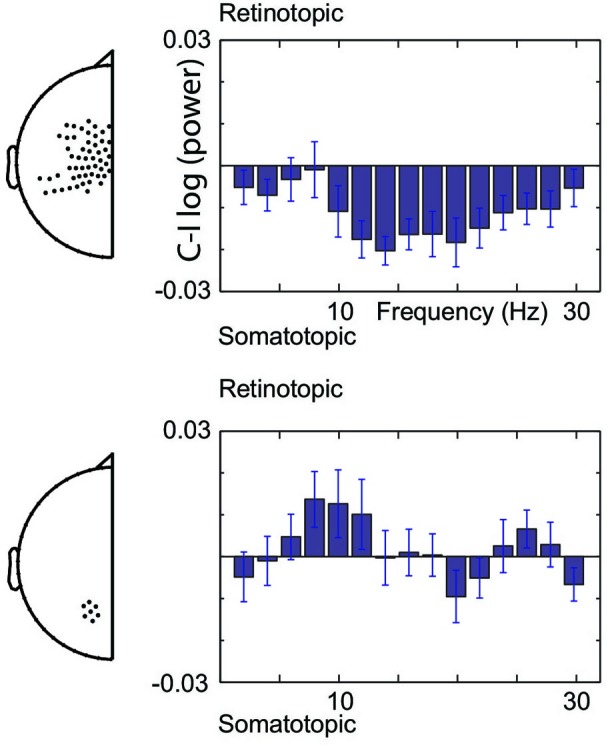
**Spectral boundaries of pre-stimulus modulations**. Gaze-centered and body-centered lateralization at central (upper) and posterior (lower) sensors under the assumption of increased excitability by alpha and beta band suppression.

Figure 3 illustrates the spectral extent of the spatially specific alpha and beta band effects. Thus, at central sensors, beta band power is relatively lower for contralateral than ipsilateral hand stimuli, whereas the alpha band shows the opposite pattern at posterior sensors. Under the assumption that suppressed alpha and beta corresponds to increased excitability (see Section Materials and Methods), the beta band modulations are consistent with increased excitability of the somatotopic areas that will be engaged. In contrast, alpha band modulations observed at posterior sensors are consistent with regulating excitability of areas in a gaze-centered reference frame. Taken together, our data show that modulations do not only take place at the sensory level, but that the brain calculates the sensorimotor transformation in anticipation of a sensory event, modulating excitability at the level of the gaze-centered oculomotor structures.

It is important to realize that our analysis of lateralization cannot distinguish which hemisphere has caused the effect. In other words, the lower power contralateral than ipsilateral could equally be due to an ipsilateral power increase or a contralateral power decrease. In the following section, we will investigate the hemisphere-specific contributions to sensorimotor behavior, by examining the correlations with saccadic reaction time.

### Prestimulus modulations correlate with saccade reaction time

If the observed power modulations indeed gate upcoming sensorimotor processing at sensory and motor stages by changing excitability of the cortical pathways, we should observe facilitating effects on saccade behavior. To test this, we correlated the pre-stimulus power modulations during the valid trials with the changes in reaction times of the correctly-directed saccades.

**Figure 4:**
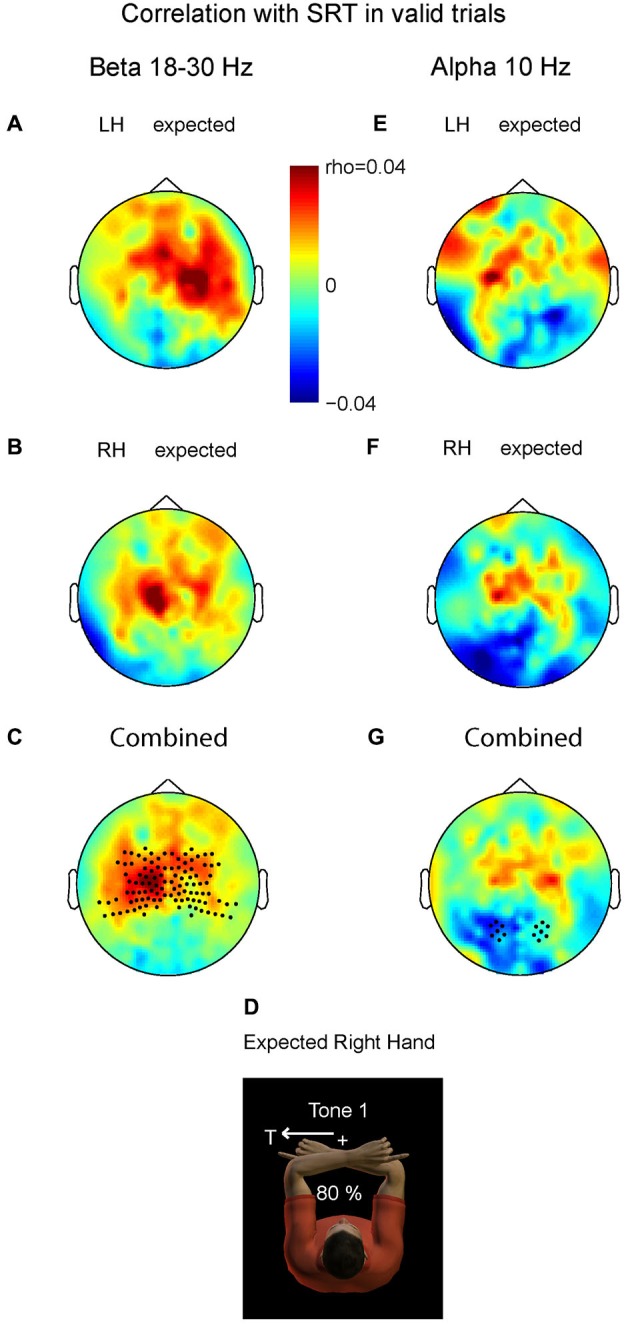
**Relationship between prestimulus power (beta and alpha band) and saccadic reaction time (SRT)**. Plotted are correlation values based on correct saccades in the valid trials. **(A)** Prestimulus beta. Left hand stimuli. **(B)** Prestimulus beta. Right hand stimuli. **(C)** Combined data. Correlation values differ significantly at central sensors between contralateral and ipsilateral hemispheres, mainly caused by positive correlation values contralateral, i.e., lower beta power for shorter SRTs. Correlation values do not differ at posterior sensors. **(D)** Depiction of expected stimulation corresponding to the format in **C** and **G**. **(E–G)** Prestimulus alpha; same format as in panel **A–C**. The more alpha ipsilateral to the saccade, the shorter its SRT. Correlation values do not differ at central sensors.

Figure 4 demonstrates the correlation values between changes in beta band power and changes in SRT for valid trials with LH stimuli (A) and valid trials with right hand stimuli (B). In both there is a small but positive correlation between the beta power at the contralateral central area and the SRT. Consistent with the inverse relationship between beta band power and cortical excitability, the more beta band suppression at contralateral central sensors, the higher the excitability and the faster the saccade is initiated (or the higher the beta, the slower). We pooled the data of both conditions by averaging the right-hand pattern with the mirrored pattern corresponding to left-hand stimulation. Figure 4C shows pooled power-SRT correlation values, in a format that renders left hemisphere contralateral and the right hemisphere ipsilateral to the stimulated hand (depicted in D). The correlation values at contralateral central sensors were significantly higher than their ipsilateral counterparts (*t* = 2.90, *P* = 0.009). This suggests that the ipsilateral hemisphere (here right) does not cause the behavioral benefits, but that the suppressed beta band power in contralateral areas is associated with expediting behavior. Correlation values at posterior sensors did not differ between hemispheres (*P* > 0.1).

Do the alpha band modulations show a similar relationship with saccade reaction time (SRT)? We performed the same analysis as for the beta band. Averaged alpha-SRT correlation values for the expected LH stimuli and right hand stimuli were small, as shown in Figures 4E,F. Figure 4D depicts the combined pattern. Correlation values differed significantly between the two hemispheres at posterior sensors (*t* = −2.32, *P* = 0.03), but not at central sensors (*P* > 0.1). Even at the source level, taking the voxel with maximum power, the central effect was not significant, ruling out that spatial summation at sensor level of central and posterior alpha sources has obscured such an effect (*P* > 0.1).

This significant difference at the posterior region was mainly caused by negative correlation values over the hemisphere contralateral to the stimulated hand. Importantly, this is the hemisphere that is ipsilateral to the direction of the required oculomotor response, and needs to be disengaged in an oculomotor reference frame. The more alpha power contralateral to the hand, and thus ipsilateral to the direction of the saccade, the shorter SRTs (or the less alpha band power the slower). These data suggest that the brain has a behavioral benefit of inhibiting gaze-centered oculomotor areas that should not become activated by premature, default intrahemispheric remapping of stimulus information.

## Discussion

We examined alpha and beta oscillations in the brain of human subjects anticipating a complex sensorimotor mapping: speeded saccades to tactile stimuli in a crossed arm posture. Our analysis was based on the increasing evidence that suppression of these oscillations is associated with higher cortical excitability (Gilbertson et al., 2005; Romei et al., 2008, 2010; Engel and Fries, 2010; Haegens et al., 2011b; Jensen et al., 2012; van Ede et al., 2012) and a recent study about the underlying reference frames of these rhythms (Buchholz et al., 2011). Behavioral data from this task suggest that early response conflict arises due to premature remapping of the tactile stimulus toward the oculomotor structure in the same hemisphere, preceding the interhemispheric remapping that is required because of the crossed arm posture (Overvliet et al., 2011; Buchholz et al., 2012). Here, we show that the brain anticipates this remapping by presetting excitability in both somatotopic (sensory) and retinotopic (motor) reference frames, as reflected by the spatial selectivity in the alpha and beta band. The positive correlation between beta band activity in the somatosensory area and SRT is consistent with somatosensory gating by beta. Conversely, alpha band activity in irrelevant oculomotor regions correlated negatively with SRT, indicating that alpha band activity gates the sensorimotor transformation by inhibition of interfering areas. Even though the correlation values and modulations were small, they show a clear topological difference between the two frequency bands. Importantly, our results were observed with predictive cues that were valid for 80%, so they contain the risk that the sensory event happens elsewhere and movement plans have to be inhibited. This could also explain the small effect sizes in comparison with other studies without such manipulations.

The slightly shorter SRTs for expected than unexpected stimuli indicate that we successfully manipulated stimulus expectation in our paradigm. Furthermore, low error rates and the size of the effect suggest that subjects used sensory evidence to drive their response and not just executed preprogrammed responses. Conversion of unexpected tactile stimuli into gaze-centered coordinates takes more than 100 ms (Heed and Röder, 2010). Our results suggest that this remapping is expedited by expectation through anticipatory neuronal population dynamics. That is, the posterior alpha band lateralization was not the same as at central regions, in contrast to intrahemispheric co-modulations observed previously (Bauer et al., 2012). The gaze-centered modulations at posterior sensors take the current eye-hand configuration into account.

Lateralization of power in the alpha band has been observed during visual (Thut et al., 2006; Hanslmayr et al., 2007; Mazaheri et al., 2009; van Dijk et al., 2010) and tactile paradigms (Haegens et al., 2010, 2011a), linking alpha band activity to modulation of cortical excitability. Scrutiny of the alpha band at central sensors suggests that it co-modulates locally with the beta band in a somatotopic manner. Moreover, across hemispheres, alpha power at central sensors did not correlate differently with SRT. This might be surprising given previous results on (dis)-engagement of somatosensory regions by alpha oscillations (Haegens et al., 2010, 2012; Jones et al., 2010; Anderson and Ding, 2011; van Ede et al., 2012). However, some of these studies used distractors on the opposite side, suggesting a specific role of alpha oscillations in functional gating by inhibition of distractor-related activity. On the other hand, alpha band activity might also be behaviorally relevant by disengaging regions that would become co-activated due to anatomical connections between regions, for example between left and right primary sensory areas (Jensen and Mazaheri, 2010).

While the observed difference in trial-to-trial power-SRT correlations were significant, they only explained a small fraction of the variance in the respective relationships (*r* ~ 0.05). Because these effects refer to condition specific differences in correlation values, the size of effect is not expected to be high (also for the reasons indicated above). Even though quantitative inferences based on extracranial recordings are limited due to methodological constraints (van Ede et al., 2012), the topography of these correlations and their polarity provide an essential insight from a functional perspective. They indicate that the brain computes its predictions about future events not only in the reference frame of the stimulus, but also simulates the coordinate transformation to anticipate the processing at the motor level.

Here, we did not use somatosensory distractors, but the sensorimotor transformation of our task contains early interference or competition from motor activity at the wrong side induced by sensory input that is not yet integrated with postural information (Overvliet et al., 2011; Buchholz et al., 2012). Therefore, the difference in correlation values observed between hemispheres was driven by negative correlation values ipsilateral, not contralateral, to the target in gaze-coordinates. Higher alpha band power in the oculomotor structures that should not become activated by early (erroneous) sensorimotor mapping was associated with shorter SRTs. This is consistent with an inhibitory role of alpha in the gating of the interhemispheric remapping process. That is, the default, but erroneous intrahemispheric remapping here might be prevented through inhibition by alpha oscillations.

Not only the alpha band at oculomotor regions expedited behavior in this task. We observed that the expectation of a somatosensory event leads to lateralization of beta band activity in central regions that is consistent with somatotopic anticipation, independent of posture. Furthermore, we found positive correlations between beta oscillations in S1 and saccadic reaction time. The difference in the correlation values across the two hemispheres was driven by positive correlations in the hemisphere contralateral to the hand. The lower the beta band power in S1 contralateral to the upcoming stimulus, the higher the excitability of this region, and the faster the saccade responses are initiated. This suggests that the local beta band power, rather than the balance between hemispheres, influences SRT.

In addition to earlier reports of behavioral benefits by beta band suppression on subsequent tactile processing (van Ede et al., 2011, 2012; Haegens et al., 2012), beta band modulations are also associated with eye-movement planning, spatial attention (Donner et al., 2007; de Lange et al., 2008; Zhang et al., 2008; Buschman and Miller, 2009; Gregoriou et al., 2012) and the facilitation of movements (Gilbertson et al., 2005). In the present paradigm, beta oscillations seem to gate only somatosensory processing and not the saccadic motor output by fronto-parietal regions.

Finally, could our results be simply explained by attentional modulations? According to the premotor theory of attention (Rizzolatti et al., 1987) preparing a saccade involves similar processes as orienting selective attention, regardless of whether the saccade is subsequently executed or not. We consider it entirely plausible that also spatial attention to the stimulus was involved, even though only foveal visual input was available. Although saccades were studied in our task, the observed gaze-centric motor code might also be part of a supramodal spatial attention network, which is activated during attentional orienting in tactile space, without explicit eye movement planning. Indeed, previous findings indicate the use of a spatial code external to a somatotopic format during tactile attention (Kennett et al., 2001; Heed and Röder, 2010). Along these lines, tactile attention might be supported by several spatial maps in parallel, prioritizing the stimulus on multiple scales, to optimally prepare the system for multisensory inputs and flexible behavioral output. In support, a recent study by Ruzzoli and Soto-Faraco (2014) showed that stimulation of parietal cortex at alpha frequencies influenced tactile attention in external space coordinates, using a task in which no saccades were involved. From a different perspective, however, the source reconstruction here matches with previous alpha band sources during saccade planning as opposed to reach planning. Alpha band activity was confined to pIPS during saccade planning (Buchholz et al., 2011); an additional source in anterior intraparietal sulcus (aIPS) has been observed during reach planning (Buchholz et al., 2013). These observations also fit nicely with imaging work showing a gradient from anterior to posterior IPS for reaches vs. saccades and proprioceptive vs. visual targets (Filimon et al., 2009). Following this reasoning, tactile attention without saccade planning might activate an intraparietal source more anterior to what we observed here. Only future work can verify this interpretation. We believe that our results demonstrate oscillatory mechanisms that could gate remapping across regions needed for both directing attention to multimodal input and preparation of potential motor acts. Via oscillatory activity, the brain could gate information flow throughout the sensorimotor network, presetting excitability of regions in this pathway that operate with different frames of reference.

## Conflict of interest statement

The authors declare that the research was conducted in the absence of any commercial or financial relationships that could be construed as a potential conflict of interest.

## Footnotes

^1^http://fieldtrip.fcdonders.nl/

^2^http://www.fil.ion.ucl.ac.uk/spm
